# Dental caries and associated factors among primary school children in Bahir Dar city: a cross-sectional study

**DOI:** 10.1186/1756-0500-7-949

**Published:** 2014-12-23

**Authors:** Wondemagegn Mulu, Tazebew Demilie, Mulat Yimer, Kassaw Meshesha, Bayeh Abera

**Affiliations:** Department of Medical Microbiology, Immunology and Parasitology, College of Medicine and Health Sciences, Bahir Dar University, Bahir Dar, Ethiopia; Department of Dentistry, College of Medicine and Health Sciences, Bahir Dar University, Bahir Dar, Ethiopia

**Keywords:** Dental caries, Dental plaque, Children

## Abstract

**Background:**

Dental caries is the most common chronic infectious disease of childhood caused by the interaction of bacteria, mainly *Streptococcus mutans* and sugary foods on tooth enamel. This study aimed at determining the prevalence and associated factors of dental caries among primary school children at Bahir Dar city.

**Methods:**

A school based cross-sectional study was conducted at Bahir Dar city from October 2013 to January 2014. Systematic random sampling technique was used to select the children. Structured questionnaire was used to interview children and/or parents to collect socio demographic variables. Clinical dental information obtained by experienced dentist using dental caries criteria set by World Health Organization. Binary and multiple logistic regression analysis were computed to investigate factors associated with dental caries.

**Results:**

Of the 147 children, 82 (55.4%) were girls. Majority of the children (67.6%) cleaned their teeth using traditional method (small stick of wood made of a special type of plant). The proportion of children having dental caries was 32 (21.8%). Primary tooth decay accounted for 24 (75%) of dental caries. The proportion of missed teeth was 7 (4.8%). The overall proportion of toothache and dental plaque among school children were 40 (27.2%) and 99 (67.3%), respectively. Grade level of 1–4 (AOR = 3.9, CI = 1.49 -10.4), poor habit of tooth cleaning (AOR = 2.6, CI = 1.08 - 6.2), dental plaque (AOR = 5.3, CI = 1.6 - 17.7) and toothache (AOR = 6.3, CI = 2.4 – 15.4) were significantly associated with dental caries.

**Conclusion:**

Dental caries is a common public health problem in school children associated with poor oral hygiene, dietary and dental visit habits. Therefore, prevention measures such as health education on oral hygiene, dietary habits and importance of dental visit are obligatory for children.

## Background

Dental caries is the most prevalent and chronic oral disease particularly in childhood age [1, 2]. Dental caries is a progressive infectious process with a multifactorial etiology [3, 4]. Dietary habits, oral microorganisms that ferment sugars, and host susceptibility have to coexist for dental caries to initiate and develop [3–5]. Dental caries has high morbidity potential. Thus, it has been the main focus of dental health professionals [6].

Dental caries is caused by dental plaque deposits on the tooth surface [7, 8]. After intake of fermentable carbohydrates, *Streptococcus mutans* undergo fermentation and produce copious amount of acid and lowers the local pH to a level where the minerals of enamel and dentine dissolve [3, 5, 7–11]. The frequent intake of sweets, dry mouth, and poor oral hygiene increase the chances for cavities [8, 12].

Dental caries causes teeth pain, discomfort, eating impairment, loss of tooth and delay language development. Furthermore, dental caries has effects on childern’s concentration in school and a financial burden on the families [6, 13]. Risk factors such as sex, age, dietary habits, socioeconomic and oral hygiene status are associated with increased prevalence and incidence of dental caries in a population [14].

Although, the trend is not clear in developing countries, the burden of dental caries has been increasing among children due to the unlimited consumption of sugary substances, poor oral care practices and inadequate health service utilization [15]. Studies revealed that the prevalence of dental caries was higher among urban children [14, 16]. Similarly, a study conducted in Ethiopia was reported 36.5% prevalence of dental caries among urban children in school [8]. Although, dental caries is more prevalent in school children, there was no documented data on prevailing prevalence and associated factors in primary school children in Bahir Dar city. Therefore, the present study was carried out to determine the prevalence and associated factors of dental caries among primary school children.

## Methods

### Study design and area

A school based cross-sectional study was conducted from October 2013 to January 2014 among primary school children at Bahir Dar city. Bahir Dar city is 560 kilometers away from the capital city, Addis Ababa. According to 2011 Central Statistical Agency of Ethiopia (CSA) estimates, the city has a total population of 220,344. Urban primary school children under 16 years accounted for 27,511. Of these, 20,340 were from public schools. The town has a total of 35 urban primary schools. Of these, 22 were private and the rest 13 were public schools [17].

### Study participants

Children from primary schools between 6 to 15 years of age and living at Bahir Dar city were included in the study. Children with 16 years of age and above, and those from private schools were excluded from the study.

### Sample size and sampling

Sample size was calculated using single population proportion formula with an assumption of 95% confidency level, 7% degree of precision and proportion of dental caries, 36.5% [8] to make the final sample size of 180. However, only 147 students provided a complete response. Systematic random sampling technique was employed to select the study participants. Among thirteen government schools, three were selected using systematic random sampling technique. The sample size was allocated proportionally based on the number of children in each selected school. Children were selected randomly based on their name lists taken from their rosters in respective class.

### Data collection

A structured questionnaire was used to collect socio-demographic characteristics, dietary habits, oral health problems and oral care practices. Dental examination was carried out for all selected children by one trained dental doctor using World Health Organization (WHO) dental caries diagnosis guide line under natural day light [15]. Disposable wooden spatulas were used for intraoral examination. Prior to the study, data collectors were given for two days intensive training on dental caries assessment based on WHO guide line and on how to interview children and fill the questionnaire. Incomplete questionnaires were rejected during data analysis. Dental caries was recorded as being present when a lesion in a pit or fissure or on smooth tooth surface had a detectable softened floor, undermined enamel or softened wall. When any doubt existed, dental caries was not recorded as present. Tooth was considered missing because of caries if a person gave a history of pain and/or presence of cavity prior to extraction.

The presence of dental plaque was assessed by direct visual inspection and palpation of the buccal and lingual surfaces of all teeth with clean glove and spatula [18]. Plaque was recorded as being present when visible deposits were detected and then removed following palpation of the teeth by clean gloved hand. Moreover, the presence of both hypo calcification and incipient caries type of white spot lesions were examined by conventional diagnostic technique [18]. First, the wet teeth were inspected for the presence of hypo calcification type of white spot lesion then the teeth were allowed to wipe cleaned and dried with gauze and compressed air to inspect incipient caries type of white spot lesion. White spot lesion was recorded as being present when a white chalky appearance spot were revealed either in dehydrated or desiccated or both type of the upper and lower anterior of enamel.

### Data analysis

Data was entered and analyzed using statistical package for social science (SPSS) version 20. Frequency and percentage were computed from univariate analysis to get summary values. Odds ratio with 95% confidence interval (CI) was computed using logistic regression analysis to assess the presence and degree of association between dental caries and independent variables. Significance was set at p < 0.05 (significance level 95%). For those variables that had a p-value < 0.05 on binary logistic regression, binary multiple logistic regression analysis was computed.

### Ethical considerations

Ethical clearance was obtained from ethical review committee of College of Medicine and Health Sciences, Bahir Dar University. A written consent was obtained from children’s parents before interview and dental examination. Cases of dental caries were advised to attend the nearby dental clinic.

## Results

### Sociodemographic characteristics

A total of 147 children were participated in the study. Of these, 82 (55.4%) were girls. The majority of children (69.6%) were from 11 to 15 years of age. Nearly half of the study participants were grade 1–4. Twenty one (14%) of the students’ parent had an education above grade 12. Eighty (54.1%) of the participants family earned below 1000 Ethiopian birr per month (Table 1).

**Table 1:** **Prevalence of dental caries and socio-demographic characteristics amon** **g primary school children at Bahir Dar city, 2014 (n = 147)**

Socio-demographic variables		Dental carries		COR (95% CI)	P- value
	Positive N (%)	Negative N (%)	Total N (%)		
**Age in years**					
6 -10	15 (33.3)	30 (66.7)	45 (30.4)	0.4 (0.18 - 1.0)	0.03
11 -15	17 (16.7)	85 (83.3)	102 (69.6	^1^	
**Sex**					
Boys	12 (18.5)	53 (81.5)	65 (44.6)	^1^	
Girls	20 (24.4)	62 (75.6)	82 (55.4)	0.7 (0.31 - 1.57)	0.87
**Grade**					
1- 4	23 (31.9)	49 (68.1)	72 (49)	0.26 (0.07 - 0.92)	0.03
5- 8	9 (12.2)	65 (87.8)	75 (51)	^1^	
**Family income**					
<1000	21 (26.3)	59 (73.8)	80 (54.1)	0.23 (0.07- 0.72)	0.2
1001-2000	5 (11.9)	37 (88.1)	42 (28.4)	0.24 (0.63 - 8.66)	0.82
>2000	6 (24)	19 (76)	25 (17.6)	^1^	
**Family educational status**					
Illiterate	10 (18.5)	44 (81.5)	54 (36.7)		
Can read and write	7 (38.9)	11 (61.1)	18 (12.2)		0.03
1-8 grade level	7 (31.8)	15 (68.2)	22 (15)		
9-12 grade level	8 (25)	24 (75)	32 (21.8)		
>12 grade level	0	21 (100)	21 (14.3)		
**Total**	**32 (21.8)**	**115 (78.2)**	**147 (100)**		

Key: COR (crude odds ratio), CI (Confidence interval), ^1^(Reference category).

### Food consumption pattern, dietary habits and practices related to oral hygiene

One hundred four (70.7%) of the children had breakfast bread with tea. Most of the children (85%) usually drank tea with sugar. Thirty one (21.1%) of the participants drank coffee with sugar. Fifty five (37.4%) of the children drank soft drinks. Seventy one, (48.3%) of the children used to eat sweet foods. One hundred five (71.4%) of the children were used to clean their teeth. Of whom, 16 (15.2%) cleaned their teeth before and after meal. However, nearly half of the children cleaned their teeth only after meal intake. Majority of the children (67.6%) used a traditional small stick of wood (termed as Mafaqiya) made of a special type of plant to clean their teeth. However, 18 (9.5%) and 5 (4.8%) were used teeth brush with and without paste, respectively to clean their teeth (Table 2).

**Table 2:** **Food consumption pattern, dietary habits and practices of oral hygiene among primary school children at Bahir Dar city, 2014**

Variables	Dental caries			P-value
	Positive N (%)	Negative N (%)	Total	
**Consumption of sugared tea (n = 147)**				
Yes	26 (20.8)	99 (79.2)	125 (85)	0.33
No	6 (27.3)	16 (72.7)	22 (15)	
**Consumption of sugared coffee (n = 147)**				
Yes	6 (19.4)	25 (80.6)	31 (21.1)	0.81
No	26 (22.6)	90 (73.4)	116 (78.9)	
**Type of food for breakfast (n = 147)**				
Bread with tea	24 (23.1)	80 (76.9)	104 (70.7)	0.47
Pasta	7 (21.1)	28 (78.8)	35 (23.8)	
Other (Makorony, Rice)	0	8	8 (5.4)	
**Consumption of sweet foods (n = 147)**				
Yes	21 (29.6)	50 (70.4)	71 (48.3)	0.03
No	11 (14.7)	64 (85.3)	76 (51.7)	
**Consumption of soft drinks (n = 147)**				
Yes	15 (27.3)	40 (72.7)	55 (37.4)	0.21
No	17 (18.5)	75 (81.5)	92 (62.6)	
**Frequency of taking soft drinks (n = 55)**				
<4/day	11 (22)	39 (76.5)	50 (91.1)	0.02
>4/day	3 (75)	1 (25)	5 (7.3)	
**Cleaning teeth ( n = 147)**				
Yes	17 (16)	88 (84)	105 (71.4)	0.016
No	15 (36.6)	26(63.4)	41 (27.9)	
**Time of tooth cleaning (n = 105)**				
Before meal	5 (16.7)	25 (83.3)	30 (28.6)	0.63
After meal	7 (13.2)	46 (86.8)	53 (50.5)	
Before and after meal	3 (18.8)	13 (81.2)	16 (15.2)	
No fixed time	2 (33.3)	4 (66.7)	6 (5.7)	
**Way of cleaning teeth (n = 105)**				
Tooth stick	10 (14.1)	61 (85.9)	71 (67.6)	0.52
Tooth brush without paste	0	5	5 (4.8)	
Tooth brush with paste	5 (27.8)	13(72.2)	18 (9.5)	
Charcoal	0	1	1 (0.96)	
Rinse with water	1 (25)	3(75)	4 (3.8)	
Other means	1 (16.7)	5(83.3)	6 (5.7)	

Key: N (number), % (percent).

### Dental caries

Of the 147 study participants, 32 (21.8%) had decayed tooth. The proportion of dental caries was 33.3% in children from 6 to 10 years of age. Girls were with a higher proportion of dental caries (24.4%) than boys (18.5%). However, the difference was not statistically significant (P = 0.87). The proportion of dental caries was 23 (31.9%) and 9 (12.2%) among children from grade1- 4 and 5–8, respectively. Children belonging to the lowest income group had the highest proportion of dental caries but the highest income group had higher prevalence than the middle income group (Table 1). Among the total dental caries, the majority 24 (75%) had primary tooth decay. Of the children who had dental caries, 12 (50%) had more than one affected tooth and 7 (21.9%) revealed missed teeth. Toothache and white spot lesions were found in 40 (27.2%) and 12 (8.2%) of children, respectively. However, dental plaque was clinically visible in 99 (67.3%) of the children. To get treatment for dental caries, 9 children (6.1%) had consulted a dentist (Table 3).

**Table 3:** **Dental cases of primary school children aged 6–15 years, at Bahir Dar city, 2014**

Variables	Frequency	Percent
**Tooth decay/dental caries (any type) (n = 147)**		
Yes	32	21.8
No	115	78.2
**Type of tooth decayed (n = 32)**		
Decay of primary tooth	24	75
Decay of permanent tooth	8	25
**Number of primary teeth decay (n = 32)**		
1 tooth	12	50
2 teeth	6	25
3 teeth	5	20.8
4 teeth	1	4.2
**Type of missed teeth (n = 7)**		
Primary	5	71.4
Permanent	2	28.6
**Tooth ache (n = 147)**		
Yes	40	27.2
No	107	72.8
**White spot lesions (n = 147)**		
Yes	12	8.2
No	135	91.8
**Plaque accumulation (n = 147)**		
Yes	99	67.3
No	48	32.7
**Health institution / Dental visit (n = 147)**		
Yes	9	6.1
No	138	93.9

### Risk factors associated with dental caries

Based on bivariate analysis, significant association between dental caries and educational status of children’s parents was found (P = 0.03). Dental caries among children, whose parents’ education are above grade 12 were 100% times at a lower risk compared to those who had non-educated parents (Table 1). There was significant association between dental caries and grade levels of children (AOR = 3.9, 95% CI = 1.49 - 10.4). Children whose grade level 1–4 were more likely to have dental caries compared to grade level of 5 – 8. Children who did not clean their teeth were 2.6 times more likely to have caries than those who cleaned (AOR = 2.6, 95% CI = 1.08 - 6.2). Children who had dental plaque were 5.3 times more likely to have dental caries than those who had not (AOR = 5.3, 95% C I = 1.6 – 17.7). Moreover, the odds of having dental caries was significantly higher among children suffer with tooth ache than those children who had not (AOR = 6.3, 95% CI = 2.4 -15.4) (Table 4).

**Table 4:** **Factors associated with dental caries in primary school children at Bahir Dar city, 2014**

Characteristics	Dental caries		COR (95% CI)	AOR (95% CI)	P-value
	Yes	No			
**Age in years**					
6-10	15	30	0.4 (0.18 - 0.89)**	0.62 (0.2 -1.92)	0.62
11-15	17	85	^1^	^1^	
**Grade level**					
1- 4	23	50	0.3 (0.13 - 0.71**	3.9 (1.49 -10.4)	0.006
5- 8	9	65	^1^	^1^	
**Cleaning teeth**					
Yes	17	89	^1^	^1^	
No	15	26	0.35 (0.15 - 0.79)**	2.6 (1.08-6.2)	0.033
**Use of sweet foods**					
Yes	21	50	0.4 (0.18 - 0.91)**	0.46 (0.18 -1.16)	0.09
No	11	65	^1^	^1^	
**Tooth ache**					
Yes	17	23	4.5 (1.97 - 10.41)***	6.3 (2.4 - 15.4)	<0.001
No	15	92	^1^	^1^	
**Plague accumulation**					
Yes	28	71	4.3 (1.43 – 13.2)**	5.3 (1.6 - 17.7)	0.006
No	4	4	1	1	

Key: COR (Crude odds ratio), AOR (adjusted odds ratio), CI (Confidence interval).

**:P-value < 0.05, ***:0.05 < P-value < 0.001.

## Discussion

In Ethiopia, there is scarcity of data on dental caries in primary school children. In this study, dental caries is a common health problem among primary school children. The prevalence of dental caries found in the present study was comparable with a study conducted in Tanzania (17.6%) [19]. However, it was lower than studies carried out in other parts of Ethiopia (36.3- 48.1%) [8, 20], Nigeria (35.5%) [1], Nepal (52%) [21] and India (77%) [22]. The difference could be due to difference in knowledge, attitude and practices (KAPs) on oral hygiene as our study was under taken among urban school children. In addition, the lower sample size in the present study might be accounted for the difference.

As a result of shortage of dental insurance and families lacking usual source of dental care, previous studies have shown that children living in poverty have higher prevalence of dental caries [7, 23, 24] but in this study a clear association between family income and dental caries was not observed. This was comparable with a study done in Sirilanka [23]. Though, the lowest income group had highest prevalence of dental caries, the middle income group had lower prevalence than the highest income group. This discrepancy might be due to other confounding factors like dietary habits.

Children’s grade level was found to be statistically significant for dental caries, hence as their grade levels increased the chance of dental caries get reduced. This finding was also supported by similar findings [8, 25, 26]. On bivariate analysis, habit of consumption of sweet foods has significantly associated with dental caries. This finding was in agreement with a study done in Saudi Arabia [5]. This might be associated with copious acid production by cariogenic bacteria like *Streptococcus mutans* that are adherent to teeth as a result of fermentation of the sweet foods. Later the enamel of the tooth went into tooth decay [5, 26]. Moreover, poor habit of tooth cleaning was significantly associated with dental caries. Children who had cleaned their teeth revealed a lower prevalence of dental caries. It is generally true that cleaning teeth will remove away the food debris from the oral. Therefore, *Streptococcus mutans* cannot get enough nutrient and time for growth and no acid production that causes dental caries development [18, 26].

It is known that toothache is one of the major indicators of tooth decay [18, 27]. In this study, children who had toothache were 6.3 times more likely to have dental caries. This finding conforms to a study conducted in Dare Salaam [27]. Moreover, dental plaque was significantly associated with dental caries. Children having visible dental plaque were more likely to have dental caries than their free counter parts. This is also a good indicator of poor oral hygiene practices. Because, dental plaque retention increases *Streptococcus mutans* colonization and in severe cases, the loss of enamel. In other studies, children with visible dental plaque recognized as a proxy of poor tooth brushing frequency. Similar finding was reported in Tanzania [19] stating that poor oral hygiene practice was the associated factor of dental carries. Moreover, such associations were established in other studies [28, 29].

This study reported that 67.6% of children cleaned their teeth using traditional small stick of wood (Mafaqiya) for maintaining oral hygiene. In contrast, other studies showed that tooth brush and tooth paste were the most common means of maintaining oral hygiene [8, 21]. In this study, a large proportion of children had never visit to a dentist. Similar finding was reported in Ethiopia [8], Nepal [21] and Sirilanka [23].

This study has the following main limitations: Detection of dental caries using dental mirror and radiology was not possible because of lack of instruments and laboratory set up. Therefore, dental caries was identified only with clinical diagnosis. In addition, the number of study participants was lower compared to other studies. Thus, this might reduce the true magnitude of the problem.

## Conclusion

Dental caries is a common public health problem among primary school children at Bahir Dar city. Low grade level, poor oral hygiene and dietary along with lack of dental visit were the associated factors for dental caries. Therefore, health education on oral hygiene, dietary habits and dental visit should be given for children to prevent and control dental caries. Moreover, further studies including private and rural school children using all methods of diagnosis of dental caries and assessment of knowledge, attitude and practices of children and their parents on oral hygiene should be recommended.

## Authors’ information

WM is an assistant professor at College of Medicine and Health Sciences, Bahir Dar University in Medical Microbiology, BA is an associate professor at college of Medicine and Health Sciences, Bahir Dar University in Medical Microbiology and department head of Medical Microbiology, Immunology and Parasitology, MY is an assistant professor at College of Medicine and Health Sciences, Bahir Dar University in Medical Parasitology. KM is lecturer at college of Medicine and Health Sciences, Bahir Dar University in doctor of dentistry.
